# Multiple Sclerosis-Like Symptoms in Mice Are Driven by Latent γHerpesvirus-68 Infected B Cells

**DOI:** 10.3389/fimmu.2020.584297

**Published:** 2020-11-19

**Authors:** Ana Citlali Márquez, Iryna Shanina, Marc Steven Horwitz

**Affiliations:** Department of Microbiology and Immunology, The University of British Columbia, Vancouver, BC, Canada

**Keywords:** multiple sclerosis, Epstein-Barr Virus, EAE, γHV-68, environmental factors, B cells

## Abstract

Multiple sclerosis (MS) is caused by a combination of genetic and environmental factors. It is believed that previous infection with Epstein Barr Virus (EBV) plays an important role in the development of MS. Previously, we developed a murine model where latent infection with gamma herpesvirus 68 (γHV-68), a murine homolog to EBV, enhanced the symptoms of experimental autoimmune encephalomyelitis (EAE), resulting in disease that more closely resembles MS in humans. Here, we explored the conditions that were necessary for EAE enhancement. We showed that latently infected CD19^+^IgD^−^ B cells were capable of enhancing EAE symptoms when transferred from mice previously infected with γHV-68 into uninfected mice. We also observed a prevention of enhancement when B cells were depleted before infection. However, depletion after the establishment of latency only partially reduced EAE. This indicated the existence of a mechanism where B cells play an important role as antigen presenting cells (APCs) prior to EAE induction for the priming of Th1 cells. It is possible that these signals persist even after B cell depletion, strongly suggesting a paracrine signaling modulation of non-B cell APCs. These results strongly support the concept that EBV contributes to the development of autoimmunity and highlights the need for a vaccine against EBV that could limit or prevent multiple sclerosis development.

## Introduction

Multiple sclerosis (MS) is a chronic inflammatory disease of the central nervous system (CNS) that affects more than 2.2 million people worldwide (1). As with many autoimmune diseases, the etiology of MS is largely unknown, although a combination of genetic elements (2–5) and environmental factors, including vitamin D deficiency, early-life obesity, gut dysbiosis, smoking, and infections have been associated with MS development (6–11).

Previous infection with Epstein-Barr Virus (EBV) is amongst the most prominent viral infections associated with contributing to the development of MS or the worsening of MS progression (12–15). Virtually 100% of MS patients are infected with EBV, and a history of infectious mononucleosis (a syndrome of EBV infection), increases the risk of developing MS later in life (16, 17).While this evidence strongly suggests that EBV contributes to MS development, the mechanism of EBV involvement in the pathogenesis of MS has been largely elusive.

Before the introduction of B cell depletion therapies with monoclonal α-CD20 antibodies (Rituximab/Ocrelizumab), it was thought that the role of B cells in MS was limited to the generation of autoantibodies (18). CD20 is a surface molecule present throughout the maturation cycle of B cells, from pre-B cells to memory cells, but not expressed on plasma cells (19). This discrepancy has caused a surge in the study of the antigen presentation ability of B cells (19–24).

Previously, our lab described an experimental model that largely mimics MS symptoms (25). Using murine gammaherpesvirus-68 (γHV-68), a rodent homolog of EBV (26), and Experimental Autoimmune Encephalomyelitis (EAE), we have been able to show that latent infection with γHV-68 leads to a high number of CD4^+^ and CD8^+^T cells infiltrating the CNS of mice induced with EAE. These immune cells produce an overwhelming Th1 response that leads to the enhancement of EAE clinical symptoms (25). Importantly, this enhanced response to EAE requires the virus to remain latent (27). When EAE is induced during acute γHV-68 infection, the start of EAE symptoms is delayed until most of the virus is cleared and latency is established. Moreover, when mice are infected with a latency free virus, they do not show signs of EAE enhancement (27). The role of γHV-68 latency and how latently infected cells affect EAE development is not yet clearly understood.

Splenic B cells, particularly the marginal zone CD19^+^IgD^−^ B cells, which exhibit features of memory B cells, harbor most of the γHV-68 virus during long term latency (28). In humans, memory B cells are also the main reservoir of latent EBV (29). Prior work by the Speck group (28) demonstrated that a small number (approximately 1 in 700) CD19^+^IgD^−^ carry latent virus 6 weeks post-infection, these, are mature B cells, most likely memory B cells. By 6 months this number drops to 1 in 2000 CD19^+^IgD^−^ cells and is likely comparable to the number of latently infected B cells post EBV infection.

It is not clear in our model if the B cells infected with γHV-68 during latency are actively participating in the enhancement of EAE, and the mechanism of this enhancement is not well understood. Here we show that the transfer of latently infected memory B cells leads to the enhancement of EAE symptoms in uninfected mice, and that γHV-68 latency establishes a precondition where B cells are pre-programmed towards a Th1 response even before EAE induction. As a model for how EBV acts as a co-factor in MS development, we propose that latent gammaherpesvirus infection of memory B cells is sufficient to modulate the immune system. This modulation presents as a strong Th1 response which leads to the development of enhanced EAE symptoms reminiscent of MS in our model.

## Methods

### Mice and Ethics Statement

C57Bl/6 mice were obtained from the Jackson Laboratory and were bred and maintained in the animal facility at the University of British Columbia. All animal work was performed in accordance with the regulation of the Canadian Council for Animal Care. The protocol was approved by the Animal Care Committee (ACC) of the University of British Columbia (Protocols A17- 0105, A17-0184).

### Infections and EAE Induction

Mice between 8 and 10 weeks of age were infected intraperitoneally (i.p.) with 10^4^ pfu γHV-68 WUMS strain (purchased from ATCC, propagated on BHK cells) or 200 µl MEM media as a control. Mice between 3 and 8 weeks old were infected intranasally (i.n.) with 400 pfu γHV-68 WUMS strain or 15 µl PBS as a control. EAE was induced at different time points post infection by injecting 100 µl emulsified Complete Freund’s Adjuvant (DIFCO) with 200µg MOG _35-55_ (GenScript) and 400µg desiccated *Mycobacterium tuberculosis* H37ra (DIFCO) subcutaneously. Mice also received two doses of 200 ng pertussis toxin (List Biologicals) *via* i.p. injection at the time of EAE induction and then again 48 h later. EAE was assessed on a score from 0 to 5 as follows: 0, no clinical symptoms, 0.5 partially limp tail; 1, paralyzed tail; 2, loss of coordination; 2.5, one hind limb paralyzed; 3, both hind limbs paralyzed; 3.5, both hind limbs paralyzed accompanied by weakness in the forelimbs; 4, forelimbs paralyzed (humane endpoint); 5, moribund or dead.

### B-Cell Depletion

B cell depletion was performed by injecting 50µg/day of α-CD20 (clone 5D2 Genentech) i.v. for four consecutive days. Optimal depletion was confirmed by Flow Cytometry.

### Viral Quantification

DNA was extracted from total splenocytes and enriched memory B cells (CD19^+^IgD^−^negative selection) at indicated time points using either TRIzol Reagent (Invitrogen) or PureLink Genomic DNA Mini Kit (Invitrogen) following manufacturer’s instructions. qPCR analysis of DNA samples was performed using 2× Quantitect Probe Mastermix (Qiagen, USA) on the Bio-Rad CFX96 Touch™ Real Time PCR Detection system with a final volume of 20 µl. Primers, probes and gBlocks^®^ were obtained from Integrated DNA Technologies. Quantification of copies of mouse genome was done on 100 ng of DNA by using primers and probe for a region of the mouse PTGER2 gene (Forward Primer: **5′-**TACCTTCAGCTGTACGCCAC**-3′**; Reverse Primer: **5′-**GCCAGGAGAATGAGGTGGTC**-3′**; Probe: **5′-**/56-FAM/CCTGCTGCT/ZEN/TATCGTGGCTG/3IABkFQ/**-3′**) (30) and absolutely quantified by use of a standard curve using concentrations from 5 × 10^7^ copies/µl to 5 × 10^1^ copies/µl. Quantification of copies of the γ-HV68 genome was done on 100 ng of DNA by using primers and probe for a region of ORF50 (Forward Primer: **5′-**TGGACTTTGACAGCCCAGTA**-3′**; Reverse Primer: **5′- **TCCCTTGAGGCAAATGATTC**-3′**; Probe: **5′-**/56-FAM/TGACAGTGC/ZEN/CTATGGCCAAGTCTTG/3IABkFQ/**-3′**) and absolutely quantified by use of a separate standard curve using concentrations from 2 × 10^4^ copies/µl to 2 copies/µl. Samples were run using a minimum of two technical replicates and all standard curves had an R^2^ greater than 0.95. The protocol was as follows: 95°C for 15 min, 95°C for 15 s, 60°C for 1 min, repeated 50 times. Quantification of copy number was done using the CFX manager software. The ratio of virus genome copy number to mouse genome copy number was obtained using the following equation:

$$\frac{\#\text{copies}\;\text{of}\;\text{ORF}50}{\#copies\;of\;PTGER}\chi \frac{2\;copies\;PTGER2/genome}{1copyORF50/genome}(\text{simplified}\;\text{to}\;\frac{\#\;\text{copies}\;\text{of}\;\text{ORF}50}{\#\;\text{copies}\;\text{of}\;\text{PTGER}2}\times 2).$$

### Immune Cell Isolation and Flow Cytometry

Mice were euthanized 17 to 25 days post EAE induction depending on the severity of EAE in the mice. They were perfused with 30 cc of PBS, and brains, spinal cords, and spleens were isolated. A single cell suspension was generated from each organ. Immune cells from the CNS were isolated using a 30% Percoll gradient. For intracellular staining, CNS mononuclear cells, were stimulated for 4 h in DMEM (Gibco) containing 10% FBS (Gibco), GolgiPlug (BD Biosciences), 10 ng/ml PMA, and 500 ng/ml ionomycin. Antibodies for the cell surface markers were added to the cells in PBS with 2% FBS for 30 min on ice. After washing, cells were resuspended in Fix/Perm buffer (eBiosciences) for 30 to 45 min on ice, washed twice, and incubated with antibodies for intracellular antigens (cytokines and transcription factors) in Perm buffer (30 min, on ice). Fluorescently conjugated antibodies directed against CD4 (clone RM4-5), CD8 (clone 53–6.7), CD3 (clone eBio500A2), IFN-γ (clone XMG1.2), Foxp3 (clone FJK-16s), and IL-17 (eBio17B7), CD19 (clone eBio1D3), IgD (clone 11–26c), RORγt (clone AFKJS-9), T-bet (clone eBio4B10), were all purchased from eBiosciences. Samples were acquired using a FACS LSR II (BD Biosciences) and analyzed using FlowJo software (Tree Star, Inc.).

### Adoptive Transfer

Spleens from γHV-68 latently infected mice were isolated 5 weeks after initial infection, and a single cell suspension was prepared. Memory B cell enrichment was performed using a custom kit from STEMCELL Technologies that contained a combination of monoclonal antibodies, including IgD, as negative selection antibodies. After enrichment, cells were washed in blank DMEM and were adjusted to a concentration of 1 to 1.5 × 10^6^ cells/200µl. Cells were injected into naïve mice and, EAE was induced the following day as described above.

### Statistical Analysis

Two-way ANOVA followed by Bonferroni’s post-test was employed to compare EAE scores. Unpaired Student’s t-test was used for all other analyses (GraphPad Prism). A p value of < 0.05 was considered statistically significant.

## Results

### Transfer of CD19^+^IgD^−^ Cells From γHV-68 Infected Mice Can Lead to EAE Enhancement in Naïve Mice

**Figure 1:**
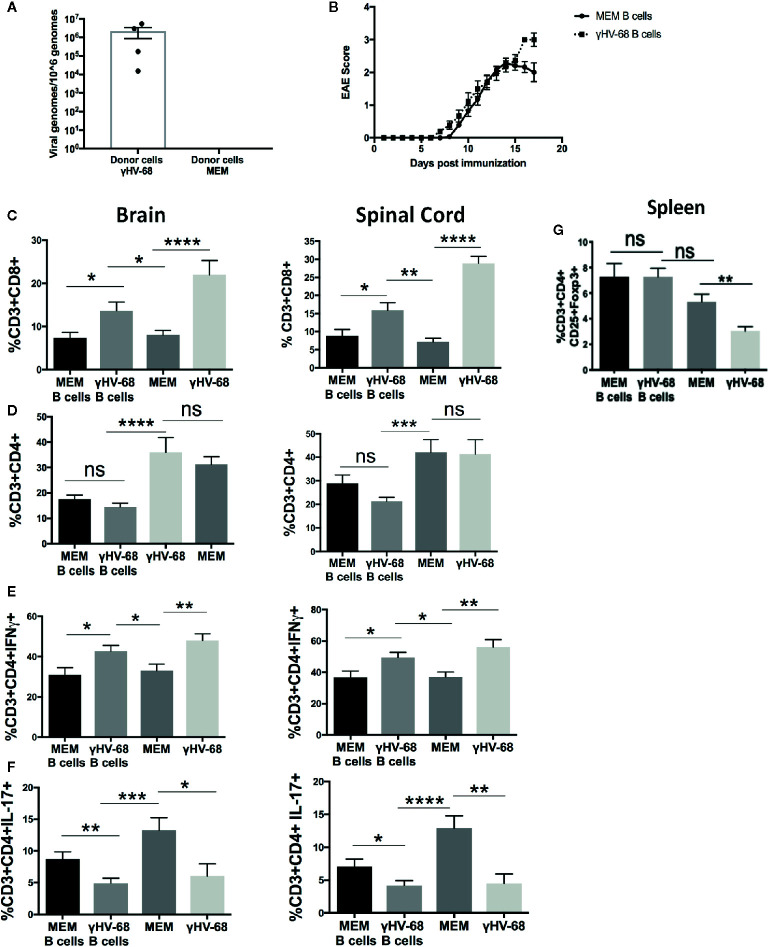
Mice were infected with γHV-68 i.p. 5 weeks p.i. spleens were harvested and CD19^+^IgD^−^ cells were enriched by negative selection. Cells were then transferred into naïve mice (γHV-68 B cells or MEM B cells). 24 h after the transfer, EAE was induced. γHV-68 latently infected mice and MEM mice were used as controls. At days 16 to 18 post EAE induction, mice were perfused; brains and spinal cords were harvested and processed to isolate immune infiltrates. **(A)** Representative qPCR showing the presence of γHV-68 in enriched CD19^+^IgD^−^ cells (no amplification in MEM cells). **(B)** Graph shows EAE scores for naïve mice that received γHV-68 B cells or MEM B cells up to day 17 post induction. **(C)** Percentage of CD8^+^ infiltrating cells in the brain and spinal cord. **(D)** Percentage of CD4^+^ infiltrating cells in the brain and spinal cord. **(E)** Percentage of CD3^+^CD4^+^IFNγ^+^ in the brain and spinal cord. **(F)** percentage of CD3^+^CD4^+^IL-17^+^ in the spinal cord. **(G)** Spleens were harvested and processed to isolate immune infiltrates. Percentage of CD3^+^ cells expressing CD4^+^CD25^+^FoxP3^+^. Three independent experiments with 6 to 24 mice/group. Data analyzed with Student’s t-test: ****p < 0. 0001, ***p < 0.001, **p < 0.01, *p < 0.05. ns = not significant.

In order to determine whether memory B cells from γHV-68 infected mice have the ability to drive EAE enhancement, we infected mice with γHV-68, waited 5 weeks for the virus to undergo latency, then isolated CD19^+^IgD^−^ cells (γHV-68 B cells) from spleen. Control mice were injected with Minimal Essential Medium (MEM) as a control (MEM B cell). We confirmed the presence of γHV-68 in γHV-68 B cells by qPCR (**Figure 1A**). No γHV-68 was detected in B cells isolated from control mice. The CD19^+^IgD^−^ B cells were adoptively transferred into naïve mice prior to induction of EAE. Previously, Willer and Speck (28) determined that CD19^+^IgD^−^ cells are the main reservoir of γHV-68 during latency, at a ratio of 1/700 infected CD19^+^IgD^−^ cells at day 42 post infection. With this in mind, we transferred 1 to 1.5 × 10^6^ cells per mouse, which would be roughly equivalent to between 1,400 and 2,000 γHV-68 infected cells per mouse. By day 17 post-EAE induction, mice that had received γHV-68 B cells reached humane endpoint and the experiment was terminated. We observed that, although not statistically significant, mice that had received γHV-68 B cells had an overall higher EAE score (average 3) than mice that received MEM B cells (average 2) (**Figure 1B**). When we compared the level of T cell infiltration into the CNS of mice receiving γHV-68 B cells compared to MEM B cells we found that there was a significant increase in CD8^+^ T cells in the brain (p < 0.05) and spinal cord (p < 0.05) of mice that received γHV-68 B cells (**Figure 1C**). Although the CD8^+^ T cell infiltration is lower than what is observed in γHV-68 infected mice, it remains significantly higher than in MEM or MEM B cells mice (p < 0.05) and is associated with increased clinical scores. Similar to what we had previously reported (25), there was no statistically significant difference in the infiltration of CD4^+^ T cells between mice that received γHV-68 B cells and MEM B cells in both the brain and spinal cord (**Figure 1D**). This confirms that CD19^+^IgD^−^ B cells from γHV-68 mice are able to drive an increase in CD8^+^ T cell infiltrates into the CNS.

### γHV-68 B Cells Increase IFNγ Production in T Cells Infiltrating the CNS

In addition to strong CD8^+^ T cell infiltration into the CNS, we have previously observed that EAE enhancement during γHV-68 latency is accompanied by a strong Th1 response, with T cells in the CNS producing high levels of IFNγ and low levels of IL-17 compared to uninfected mice (25). To test if the presence of CD19^+^IgD^−^ cells from γHV-68 mice impacts the production of these cytokines, we used flow cytometry to detect IFNγ and IL-17 in the brain and spinal cord of mice that received γHV-68 B cells versus those that received MEM B cells. We found that CD4^+^ T cells from mice with γHV-68 B cells were producing similar amounts of IFNγ in both brain and spinal cord than mice infected with γHV-68, and in quantities considerably higher than mice that received MEM B cells and MEM mice (**Figure 1E**). We also observed a marked downregulation of IL-17 produced by CD4^+^ T cells in mice that received γHV-68 B cells compared to those that received MEM B cells (p < 0.01) and MEM mice (p < 0.001) (**Figure 1F**), and at the same level as γHV-68 mice (p=ns).

One of the most significant changes during latency is the downregulation of Tregs in the periphery and CNS (27). When we induced EAE in mice that received CD19^+^IgD^−^ cells, we found that the level of Tregs in mice that received γHV-68 B cells remained the same as mice receiving B cells from uninfected mice (p=ns) (**Figure 1G**). This suggests that while CD19^+^IgD^−^ cells from infected mice are able to affect CNS infiltration and cytokine production by infiltrating cells, they do not affect the overall number of Tregs in the periphery.

Overall, these data indicate that CD19^+^IgD^−^ cells from latently infected mice are actively interacting with other immune cells and altering the production of cytokines during latency.

### B-Cell Depletion During EAE Moderately Improves EAE Score but Does Not Affect T-Cell Infiltration Into the CNS

**Figure 2:**
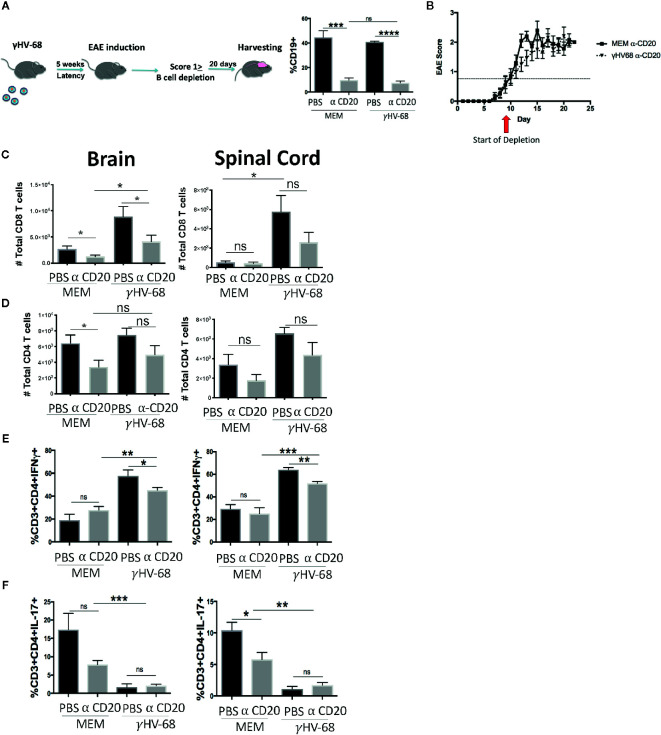
Mice were infected with γHV-68 or MEM i.p. 5 weeks p.i. EAE was induced. When mice reached a score of ≥1 B cells were depleted with α-CD20 i.v. At days 20 to 22 post EAE induction, mice were perfused; brains, spinal cords were harvested and processed to isolate immune infiltrates. **(A)** B cell depletion workflow. Histogram shows a representative experiment of B population at moment of harvesting. **(B)** Graph shows EAE scores up to day 23 post induction. **(C)** Total CD8^+^ infiltrating cells in the brain and spinal cord. **(D)** Total CD4^+^ infiltrating cells in the brain and spinal cord. **(E)** Percentage of CD3^+^CD4^+^IFN γ^+^. (**F)** CD3^+^CD4^+^IL-17^+^ in the brain and spinal cord. Three independent experiments with 6 to 11 mice/group. Data analyzed with Student’s t-test: ***p < 0.001, **p < 0.01, *p < 0.05. ns = not significant.

Although the mechanism for why B cell depletion helps stop MS relapses has not yet been identified, it has been suggested that part of its success is due to the depletion of the main reservoir of latent EBV (31). Given the strong effect of B cells in EAE (19–21, 32) we explored whether depleting B cells in γHV-68 infected mice was able to eliminate the effect that memory B cells have during EAE. We depleted B cells in mice latently infected with γHV-68 (γHV-68/α-CD20) or uninfected controls (MEM/α-CD20) that had an EAE score ≥1 with a murine α-CD20 (or PBS as control), we confirmed effective B cell depletion in splenocytes at end point (**Figure 2A**). When B cells were depleted when EAE symptoms first appeared, γHV-68/α-CD20 mice did not show improvement in their EAE score, and, by day 20, mice had an average score of 2.5 to 3 (**Figure 2B** and **Supplementary 1**), even when we observed a significant reduction of the total number of CD8^+^ T cells in the brain of γHV-68/α-CD20 mice compared to γHV-68/PBS mice (p < 0.05). This reduction of CD8^+^ T cell infiltration was not at the same level as MEM mice (p < 0.05) (**Figure 2C**), and CD4^+^ T cells remained mostly unchanged (p=ns) (**Figure 2D**). Interestingly, we saw a significant reduction in IFNγ production in CD4^+^ T cells in the brain and spinal cord of γHV-68/α-CD20 mice compared to γHV-68/PBS mice (p < 0.01) while IL-17 remained downregulated in CD4^+^ infiltrating T cells (p < 0.001) (**Figures 2E, F**). Although IFNγ was still upregulated compared to uninfected controls, this slight reduction could explain why α-CD20 mice showed a slight improvement in their EAE score right after depletion.

### Depletion of B Cells Before EAE Induction Does Ameliorate Symptoms

**Figure 3:**
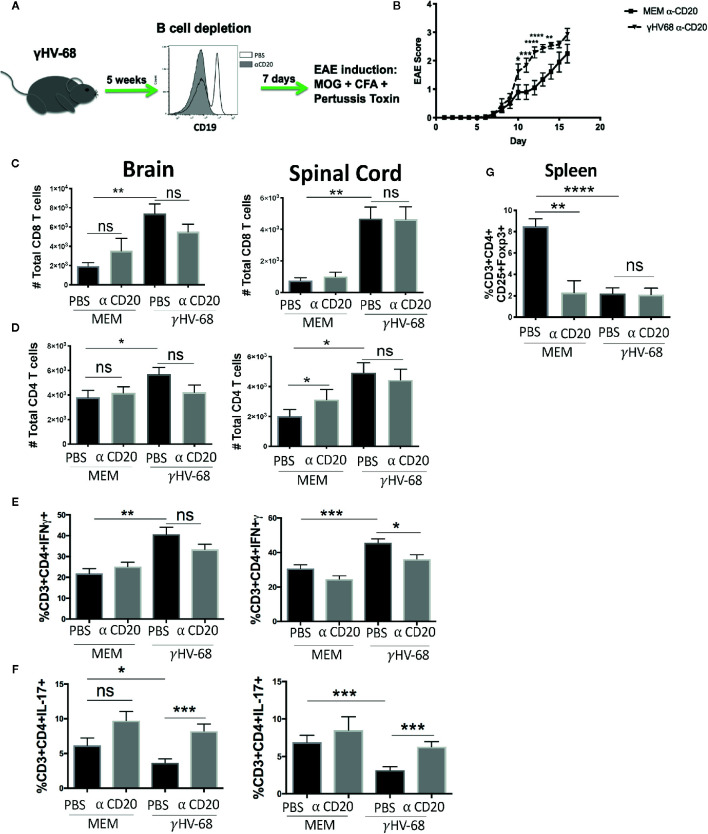
Mice were infected with γHV-68 or MEM i.p. 5 weeks p.i. B cells were depleted with α-CD20 i.v. 2 days after depletion EAE was induced. At days 21 to 23 post EAE induction, mice were perfused; brains and spinal cords were harvested and processed to isolate immune infiltrates. **(A)** Histogram shows a representative experiment of effectiveness of B cell depletion. **(B)** Graph shows EAE scores up to day 23 post induction. **(C)** Percentage of CD8^+^ infiltrating cells in the brain and spinal cord. Data analyzed with two-way ANOVA test with Bonferroni’s correction for multiple comparisons: ***p < 0.001, *p < 0.05 **(D)** Percentage of CD4^+^ infiltrating cells in the brain and spinal cord. **(E)** Percentage of CD3^+^CD4^+^IFNγ ^+^ and **(F)** CD3^+^CD4^+^IL-17^+^ in the brain and spinal cord. **(G)** Spleens were harvested and processed to isolate immune infiltrates. Percentage of CD3^+^ cells expressing CD4^+^CD25^+^FoxP3^+^. Five independent experiments 20 to 25 mice/group. Data analyzed student’s t-test: ****p < 0.0001 ***p < 0.001, **p < 0.01, *p < 0.05. ns = not significant.

In order to determine if EAE enhancement could be stopped by depleting B cells just prior to EAE induction, we infected mice with γHV-68, waited for 5 weeks p.i, and then depleted B cells with α-CD20 antibody or PBS as a control (**Figure 3A**). Scores remained higher in γHV-68/α-CD20 mice compared to MEM/αCD-20 mice (**Figure 3B** and **Supplementary 2**).

Additionally, depletion before EAE induction did not affect the infiltration of CD8^+^ T cells into the CNS (p=ns) (**Figures 3C, D**). When we tested cytokine production by these infiltrating T cells, we saw that even though a strong production of IFNγ in the brain and spinal cord remained present, there was a trend towards downregulation (p=ns) (**Figure 3E**). More significantly, we observed an upregulation of IL-17 to levels similar to the uninfected control groups (p < 0.001) (**Figure 3F**). This strongly suggests that while depleting B cells before the onset of symptoms helps to restore some of the balance in cytokine production in T cell infiltrating cells, it does not stop EAE enhancement. Yet, it may imply that latently infected memory B cells are key to both disease initiation and maintenance. Interestingly, depletion did not allow for the Treg population to recover (**Figure 3G**) in either the γHV-68 mice or MEM/αCD-20 groups (p < 0.0001). The latter group has been reported to change as a result of B cell depletion (33).

These results suggest that once EAE is initiated, removal of B cells from γHV-68 infected mice likely only affects disease severity through Th balance skewing.

### γHV-68 Latency Establishes a Th1 Precondition That Leads to the Enhancement of EAE

**Figure 4:**
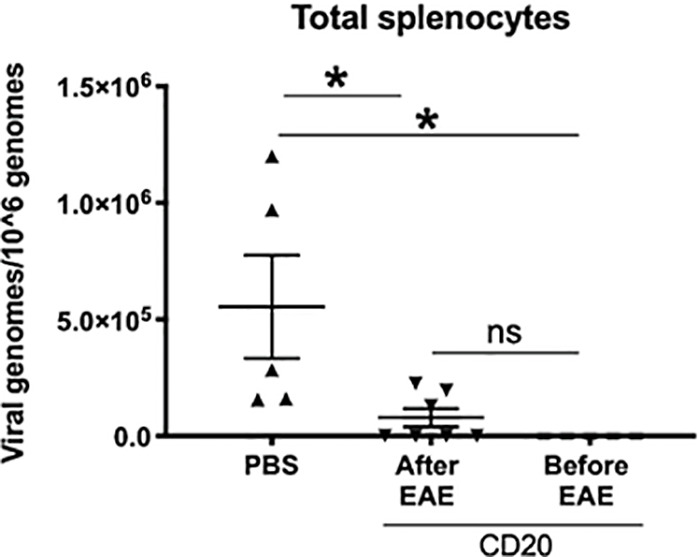
B cells from γHV-68 latently infected mice were depleted with α-CD20 i.v. at different time points; before/after EAE induction. Splenocytes were collected at experimental point and qPCR was performed to detect γHV-68. Two independent experiments with 5 to 10 mice/group. Data analyzed with Student’s t-test: *p < 0.05. ns = not significant.

The fact that B cell depletion only partially altered EAE in γHV-68 infected mice suggests that either not all the virus was cleared with depletion, and/or immune cells in latently infected mice are preprogrammed towards a strong Th1 response. In order to determine whether there was γHV-68 remaining after α-CD20 depletion, we quantified the amount of virus present in splenocytes at both points of depletion (**Figure 4**). We observed that depletion of B cells before EAE induction was highly effective, as all the samples lacked detectable virus. However, B cell depletion after the development of EAE symptoms was not completely effective and a minority of samples retained virus. This suggests that the different effects that we observed in both treatments with α-CD20 might be related to the presence/absence of latent virus and/or an effect of latency in the immune system prior to EAE induction. In other words, the effectiveness of treatment is dependent on the time point of depletion relative to the inciting event.

**Figure 5:**
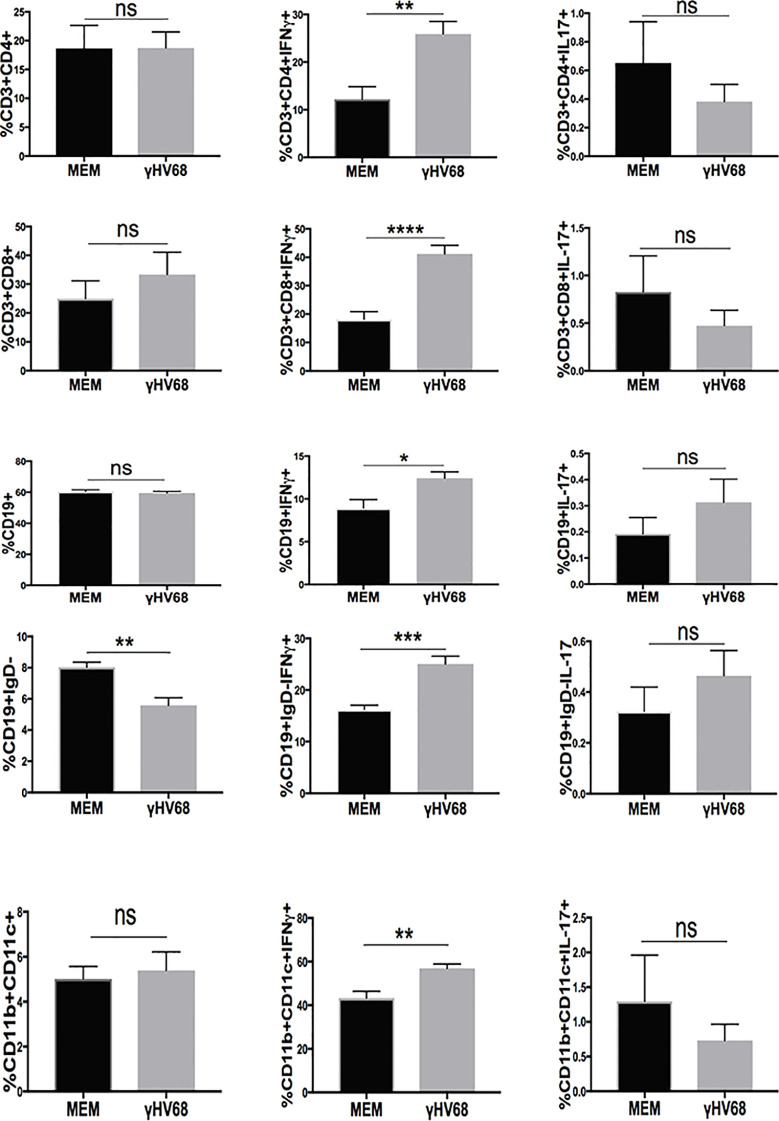
Mice were infected with γHV-68 i.p. 5 weeks p.i. spleens were harvested, stimulated with PMA/Ionomycin and stained for IFN γ and IL-17 in in CD3^+^CD4^+^, CD3^+^CD8^+^,CD19^+^, CD19^+^IgD^−^, CD11b^+^CD11c^+^ cells. Two independent experiments with 6 to 11 mice/group. Data analyzed with Student’s t-test: ****p < 0. 0001, ***p < 0.001, **p < 0.01, *p < 0.05. ns = not significant.

To test if there were any changes occurring in the immune system during latency before EAE induction, we harvested splenocytes from γHV-68 infected mice and determined their IFNγ and IL-17 production when they were unspecifically stimulated with PMA/Ionomycin. We observed that while most of the immune cells were present in the same proportion between γHV-68 and MEM mice during latency, cells from γHV-68 mice were already primed towards a Th1 response, with CD4^+^ T cells (p < 0.01), CD8^+^ T cells (p < 0.0001), CD19^+^ B cells (p < 0.05), CD19^+^IgD^−^ B cells (p < 0.001) and CD11b+CD11c+(p < 0.01) producing significantly more IFNγ (**Figure 5**). While the number of CD19^+^IgD^−^ cells decreased in γHV-68 infected mice, they are still potent IFNγ producers and are pre-programmed towards both a Th1 response themselves and to drive subsequent strong Th1 responses.

### EAE Enhancement Depends on the Presence of B Cells During γHV-68 Infection

**Figure 6:**
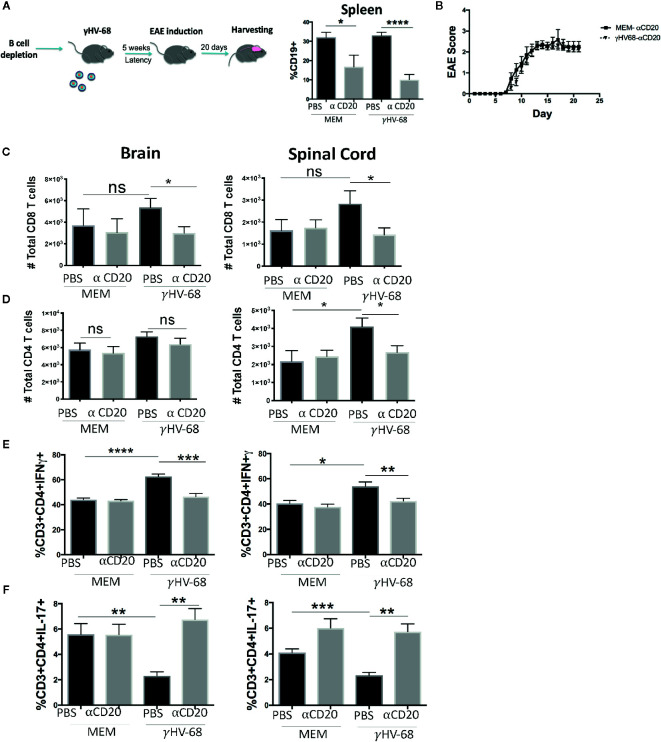
B cells were depleted with α-CD20 i.v. 2 days after depletion, mice where infected with γHV-68 or MEM i.p. 5 weeks p.i. EAE was induced. At day 21 post EAE induction, mice were perfused; brains and spinal cords and spleen were harvested and processed to isolate immune infiltrates. **(A)** Histogram shows B cell levels at experimental end point. **(B)** Graph shows EAE scores up to day 21 post induction. **(C)** Total CD8^+^ infiltrating cells in the brain and spinal cord. **(D)** Total CD4^+^ infiltrating cells in the brain and spinal cord. Two independent experiments with 6 to 12 per group. **(E)** Percentage of CD3^+^CD4^+^IFN γ^+^ and **(F)** CD3^+^CD4^+^IL-17^+^ in the brain and spinal cord. Two independent experiments with 5 to 12 mice/group. Data analyzed with Student’s t-test: ****p < 0. 0001, ***p < 0.001, **p < 0.01, *p < 0.05. ns = not significant.

The experiments described above suggest that once γHV-68 latency is established, the immune system of infected mice establishes a precondition that favors a Th1 T cell response. It is known that γHV-68 latency can be established in other cell types in the absence of B cells (34). We explored if the effect of γHV-68 in EAE enhancement was limited to the establishment of latency in B cells or if the potential establishment of latency in other cell types could also lead to EAE enhancement. To investigate this we depleted B cells prior to γHV-68 infection and then, as before, induced EAE 5 weeks after infection (**Figure 6A**). Importantly, there was no difference in EAE score between γHV-68/α-CD20 mice and the MEM/α-CD20 controls similar to what is observed in those mice that have not been infected (**Figure 6B** and **Supplementary 3**). Significantly, we observed that the level of infiltration of CD8^+^ T cells in the brain and spinal cord was similar among γHV-68/α-CD20, MEM/α-CD20 and MEM/PBS mice, with a complete reduction of CD8^+^ T cell infiltration into the CNS in γHV-68 mice (p < 0.05) (**Figures 6C, D**). Moreover, we observed similar cytokine production levels from infiltrating CD4^+^ T cells between the γHV-68/α-CD20 vs the MEM groups (p < 0.0001), while the γHV-68/PBS group lacking depletion demonstrated strong IFNγ production and a marked downregulation in Th17 (p < 0.01 and p < 0.05) (**Figures 6E, F**).

**Figure 7:**
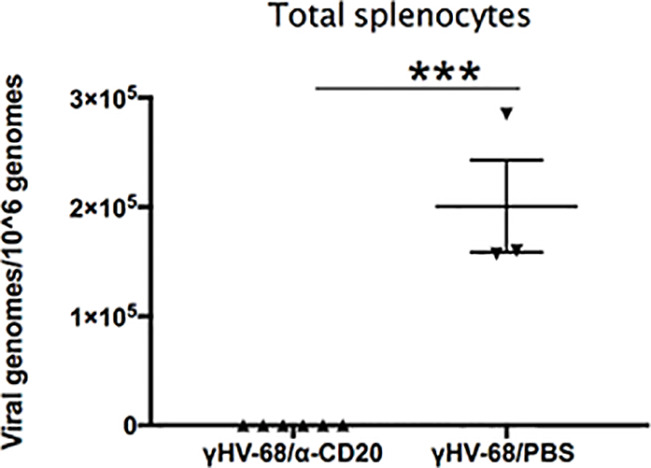
B cells were depleted with α-CD20 i.v. 2 days after depletion, mice where infected with γHV-68 or MEM i.p. 5 weeks p.i. EAE was induced. Spleens were harvested at 35 days p.i., DNA was extracted, and viral genomes were quantified by qPCR. Graph shows quantification of γHV-68 in mice depleted with α-CD20 vs PBS. Two independent experiments with 3 to 6 mice/group. Data analyzed with Student’s t-test: ***p < 0.001.

Since memory B cells are the main reservoir of γHV-68 after acute infection, we determined if the virus was able to establish latency in mice without B cells. We quantified γHV-68 in the whole spleen at 35 days p.i. (**Figure 7**). No virus was detected in the spleen of mice that underwent B cell depletion prior to infection. This is in agreement with other reports where, in the absence of B cells, the virus is less efficient in establishing latency in the spleen but does establish latency in some peritoneal cells and the lungs (34, 35). If present, the small number of latently infected cells were not able to enhance EAE. Taken together, our results clearly demonstrate the importance of latently infected B cells in driving a strong Th1 response that directly enhances EAE pathogenesis reminiscent of MS. Importantly, our results also show that for enhanced disease to develop, γHV-68 needs to establish latency in memory B cells.

## Discussion

In recent years, the unexpected efficiency of B cell depletion therapies has increased interest in the role of B cells in autoimmune diseases, and in MS in particular. To date, the role of B cells in the development of MS is not well understood, but we, and others (31, 36–38), have suggested that the link might be in the latent expression of EBV in memory B cells. Our lab has previously shown (25) that CD11b^+^CD11c^+^ cells from latently infected mice were able to direct a strong production of IFNγ. However, these cells were not infected with γHV-68 (25), which suggested that CD11b^+^CD11c^+^ cells should have been primed by infected B cells during latency.

In this paper, we demonstrated that “γHV-68 latently infected B cells” are indispensable for the enhancement of EAE. The transfer of CD19^+^IgD^−^ cells from γHV-68 mice into naïve mice showed that memory B cells were actively contributing to directing the strong Th1 response during EAE. While the depletion of B cells with an α-CD20 before and after EAE induction was unsuccessful in decreasing overall disease, we are still able to see high levels of infiltration of T cells into the CNS, and a high production of IFNγ. These results suggest that the effects of latency in the immune system can be felt even after the elimination of B cells and most of the latent virus.

Interestingly, IL-17 is one of the cytokines that changes the most depending on when depletion occurs, in both infected mice and uninfected mice. When B cell depletion occurred once EAE was induced, we saw an overall downregulation of IL-17. However, when B cell depletion occurred before EAE induction, we saw an overall recovery of IL-17 production, accompanied with a slight downregulation of IFNγ. This suggests that by eliminating most of the virus before EAE induction, the balance of IFNγ/IL-17 was partially restored. There is an interdependent relationship between B cells and Th17. For example, IL-6, a cytokine necessary for IL-17 production, is also a cytokine needed in B cell proliferation (21, 39). Similar to what we observe in B cell depletions before EAE induction, any disturbance in the B cell repertoire in humans and mice seems to also downregulate IL-17 production (40).

Ultimately, the fact that latency in B cells establishes a precondition towards a Th1 response strongly suggests that the only way to effectively eliminate the effects of latency on EAE development is by depleting B cells before primary infection in order to avoid latency in B cells. This is particularly relevant to MS therapy, since it suggests that in order to have a lasting effect from B cell depletion, initial infection should be avoided. While this is generally not possible with a ubiquitous virus like EBV, its strong link to cancer and other disorders could entertain the possibility of a childhood vaccine to prevent a number of diseases. It would be also interesting to determine whether multiple cycles of B cell depletion before EAE induction could more thoroughly wane the programming of other immune cells. Defining differential biomarkers of B cells from latently infected mice is essential to targeting disease progression. One possibility is that B cells from γHV-68 have increased antigen presentation capabilities, something that has been observed in memory B cells from MS patients (41, 42). Finally, exploring the role of type I IFNs in our model could provide important insight into what kind of signals are being produced by B cells in infected mice. One possibility is interferon-beta, which has been widely used in the treatment of Relapsing Remitting-MS patients (43), although with limited success (44). Additionally, Type I IFN production is necessary to control reactivation and maintenance of latency of γHV-68 (45) as well as in EBV (46).

In summary, we demonstrated that latently infected memory B cells are critical for driving enhanced disease reminiscent of MS and it is highly likely that EBV may be acting in similar ways in pre-MS and MS patients. We propose that treatments directed at targeting virus or latently infected memory B cells will have more success than current treatments in stopping MS development. The data on B cell depletion therapies is supportive of this notion. However, it also highlights that in order to achieve the highest level of protection against MS is avoiding infection with EBV at all. Hence, the development of a vaccine against EBV might be the only effective way to prevent the effects of EBV in Multiple Sclerosis.

## Data Availability Statement

The original contributions presented in the study are included in the article/**Supplementary Material**. Further inquiries can be directed to the corresponding author.

## Ethics Statement

The animal study was reviewed and approved by the Animal Care Committee University of British Columbia.

## Author Contributions

AM and MH conceived and designed the experiments. AM and IS conducted the experiments. AM and MH analyzed the results and wrote the manuscript. All authors contributed to the article and approved the submitted version.

## Funding

This work was supported by a grant from the MS Society of Canada to MH. AM received a PhD fellowship from the MS Society of Canada, Consejo Nacional de Ciencia y Tecnologia (CONACyT) and The American Association of Immunologists.

## Conflict of Interest

The authors declare that the research was conducted in the absence of any commercial or financial relationships that could be construed as a potential conflict of interest.
