# Supplementary material for: Multiple Sclerosis-Like Symptoms in Mice Are Driven by Latent γHerpesvirus-68 Infected B Cells
Source: Front Immunol. 2020 Nov 19;11:584297. doi: 10.3389/fimmu.2020.584297 (PMC7711133; doi:10.3389/fimmu.2020.584297)
Supplement: Supplementary file 1 [file Table_3.pdf]

Supplementary 1. Mice were infected with γHV-68 or MEM i.p. 5 weeks p.i. EAE was induced. When mice reached a score of $\geq$1 B cells were depleted with α-CD20 i.v. At days 20-22 post EAE induction, mice were perfused; brains and spinal cords were harvested and processed to isolate immune infiltrates. Graph shows EAE scores up to day 23 post induction. Three independent experiments with 6-11mice/ group. Data analyzed with two-way ANOVA test with Bonferroni's correction for multiple comparisons

Supplementary 2. Mice were infected with γHV-68 or MEM i.p. 5 weeks p.i. B cells were depleted with α-CD20 i.v. 2 days after depletion EAE was induced. At days 21-23 post EAE induction, mice were perfused; brains and spinal cords were harvested and processed to isolate immune infiltrates. Graph shows EAE scores up to day 23 post induction. Data analyzed with two-way ANOVA test with Bonferroni's correction for multiple comparisons: ***$p<0.001$, *$p<0.05$. Five independent experiments 20-25 mice group.

Supplementary 3. B cells were depleted with α-CD20 i.v. 2 days after depletion, mice where infected with γHV-68 or MEM i.p. 5 weeks p.i. EAE was induced. At day 21 post EAE induction, mice were perfused; brains and spinal cords and spleen were harvested and processed to isolate immune infiltrates. Graph shows EAE scores up to day 21 post induction. Two independent experiments with 6-12mice/ group.
