# Supplementary figures and images for: Multiple Sclerosis-Like Symptoms in Mice Are Driven by Latent γHerpesvirus-68 Infected B Cells
Source: Front Immunol. 2020 Nov 19;11:584297. doi: 10.3389/fimmu.2020.584297 (PMC7711133; doi:10.3389/fimmu.2020.584297)

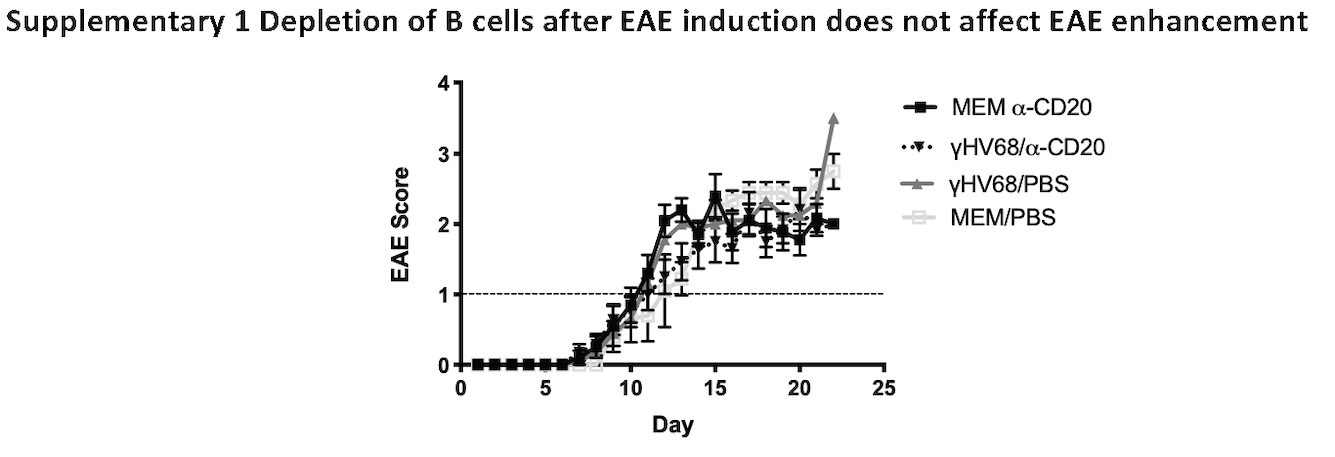

Supplement: Supplementary file 2 [file Image_1.tiff]

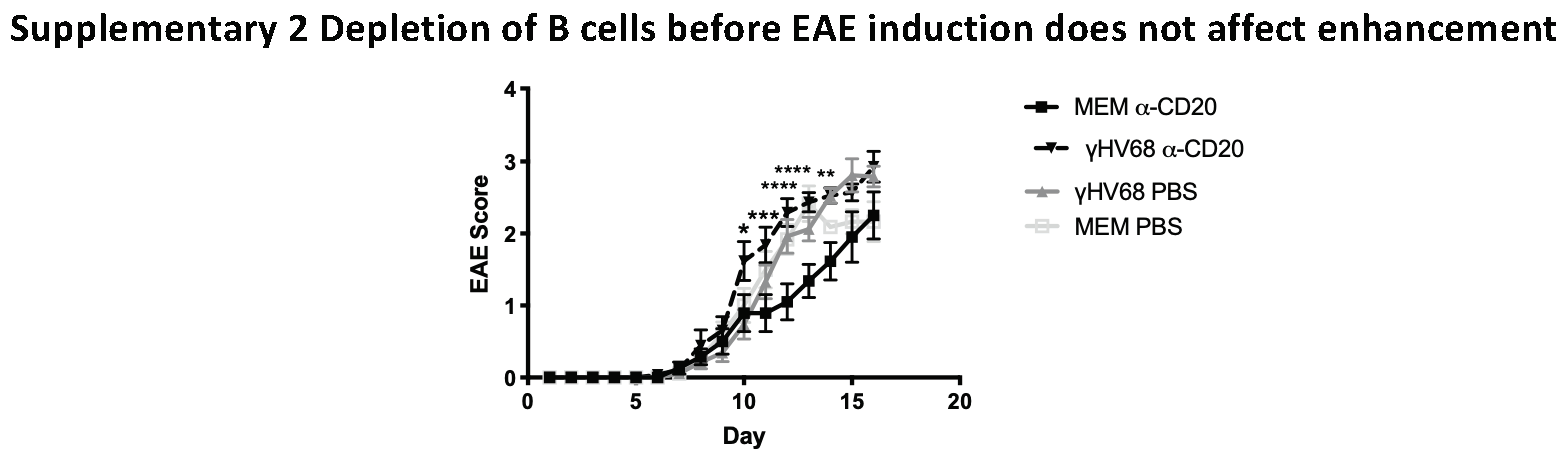

Supplement: Supplementary file 3 [file Image_2.tiff]

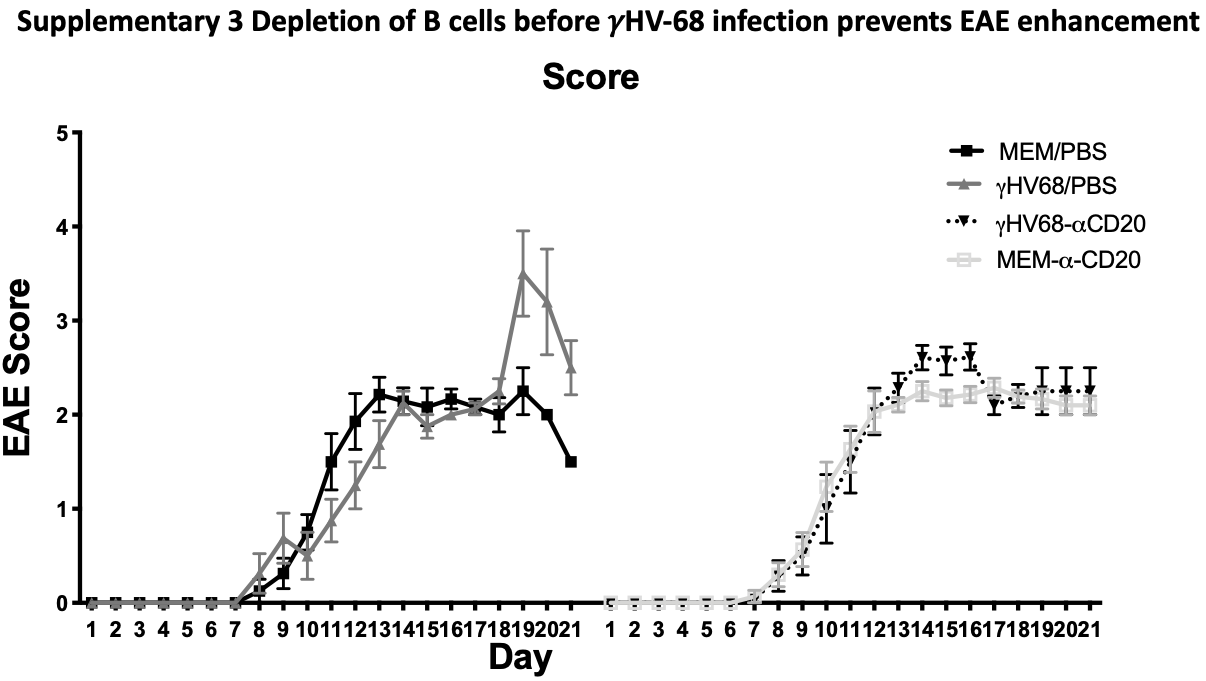

Supplement: Supplementary file 4 [file Image_3.tiff]
